# Wireless Sensor Network-Based Rockfall and Landslide Monitoring Systems: A Review

**DOI:** 10.3390/s23167278

**Published:** 2023-08-20

**Authors:** Mattia Ragnoli, Massimo Scarsella, Alfiero Leoni, Giuseppe Ferri, Vincenzo Stornelli

**Affiliations:** Department of Industrial and Information Engineering and Economics, University of L’Aquila, 67100 L’Aquila, Italy; massimo.scarsella@graduate.univaq.it (M.S.); alfiero.leoni@univaq.it (A.L.); giuseppe.ferri@univaq.it (G.F.); vincenzo.stornelli@univaq.it (V.S.)

**Keywords:** geohazard, IoT, landslide monitoring, risk assessment, rockfall monitoring, sensors, wireless sensor network

## Abstract

Rockfalls and landslide events are caused by different factors among which are included geomorphological and climatic factors and also human interaction. Therefore, the economic and social impacts can be significant and the remote monitoring of such hazards has become an essential topic in various applications. Wireless sensor networks (WSNs) are well suited for the deployment of monitoring systems, benefiting from the different technologies and topologies that are available and evolving nowadays. This review paper aims to summarize and overview the up-to-date state of the art of rockfall and landslide monitoring systems based on WSNs. The implementation and methods were analyzed for each solution, along with the system architecture and relevant hardware aspects. All the retrieved data were used to analyze the current trends and future possibilities in the field of WSN geohazard monitoring.

## 1. Introduction

### 1.1. Rockfall and Landslide Mechanisms

Geologic hazards (geohazards) are naturally occurring or, in some cases, induced by humans. These events cause damage, loss of property and, in the worst cases, pose threat to life [1]. Landslides and rockfalls are geohazards that depend on the combination of climatic and geomorphological factors [2,3]. Their triggering mechanisms are different [4,5,6], such as severe rainfall, seismic events, vegetation interaction, and human action.

The term landslide refers to a displacement of a mass of rock, sediment, or residual soil that is adjacent to a slope, in which the center of gravity of the moving mass moves down and outward. A movement which is similar but proceeds at an imperceptible rate is called creep. The velocity of the masses involved in a typical landslide increases from almost zero to at least 0.3 m per hour [7]. Rockfalls are rapid slope instability processes that can affect mountainous regions, slope cuts, and coastal cliffs. We can identify these events as a small landslide which involves the removal of individual rocks of dimensions below 5 m^3^ from a cliff face [8]. Rockfall is unpredictable, but in general it mostly occurs in the spring season. This is due to the alternance of the freezing and thawing of water [9]. The reports of geohazards can be found in official sources such as the Office for the Coordination of Humanitarian Affairs (OCHA) [10].

The prevention of riskful events is based on the application of basic geologic engineering principles and technologies to prevent, minimize or reduce the effects [11]. Rockfall- and landslide-affected scenarios have been studied in the past for various reasons. An example is the risk assessment, as it is indispensable for the management of landslide and rockfall hazards and for planning risk mitigation measures [12,13,14]. The correct study of susceptibility zones for catastrophic events is necessary for appropriate land planning [15]. 

The authors of [16] provided an assessment of global landslide susceptibility (LSS). The study was carried out at a 36 km spatial resolution of global satellite soil moisture observations, in order to prepare a combination of a global LSS map with dynamic soil moisture estimates for landslide modelling. The analysis carried out in [17] revealed the main topics, countries, and institutions that have conducted research regarding the topic of rockfall and landslides. 

### 1.2. Monitoring Systems for Geohazards

Monitoring and early warning are commonly studied and applied strategies for the prevention of geohazards. The solutions available in the literature perform well in certain tasks but are lacking in others, and it is not always clear which fits a specific application better. The main reason behind this diversity resides in the different implementation possibilities of a sensor network and in its multidisciplinary nature. Considering the subset of landslide and rockfall monitoring systems, the list of variables involved is large. Different technologies have been studied and applied to this range of variables. In situ sensor networks are employed to gather information on the geological conditions of the monitored area. 

In general, a monitoring system for geohazard prevention can be schematized as follows:

- A sensing layer, where sensors are used to gather data about a specific physical dimension relative to a geomorphological condition;
- A data exchange layer, where wired or wireless technologies are used to transport the information;
- A data storage layer, where gathered data are stored in a database for successive analysis;
- A data analysis layer, where data are analyzed to obtain early warnings, risk assessment, and generally useful information.

In the past, wired data acquisition systems were used to store data for the different applications of structural monitoring [18,19,20,21,22]. Wiring and power constraints can, in some cases, increase the overall cost and complexity of the system, thus limiting the number and locations of sensor nodes. Installation and maintenance costs may be increased too. In WSNs, the sensor nodes are deployed without wiring; thus, this technological solution offers an advantage in dealing with all the aforementioned issues. These positive aspects can be successfully applied to geohazard monitoring systems [23], leading to new, more flexible solutions. 

This paper aims to give a comprehensive overview of the scientific literature that deals with rockfall and landslide monitoring with systems based on wireless sensor networks. Over the years, the technological advances in geohazard monitoring systems have moved towards low-power and more efficient implementations. The information that has been gathered in this review can be used for the development of new systems. To this extent, the characteristics and performances of the state-of-the-art systems are provided to overcome current research limitations and embrace future trends and challenges. Section 2 reports a brief description of the state of the art related to WSNs. In Section 3, several works dealing with the aforementioned geohazard monitoring systems are reviewed, together with some other works dealing with different technologies that have been used. Section 4 reports a discussion based on the reviewed research and future perspectives. Section 5 summarizes the work carried out in this paper.

## 2. State-of-the-Art Overview of Wireless Sensor Networks for Monitoring Applications

**Figure 1:**
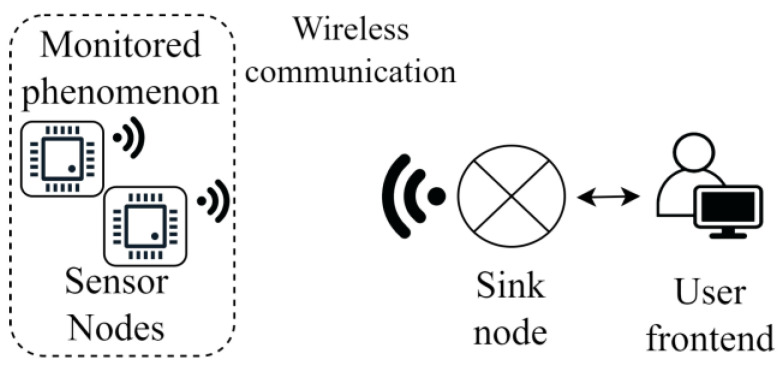
General architecture of a WSN.

In recent years, WSNs have received increasing interest from industrial and research perspectives [24,25,26]. In a WSN, a group of nodes forms a network where the elements sense and may control the environment. The data is exchanged through a gateway to a sink via single or multiple hops. Figure 1 shows the general architecture of a WSN monitoring network.

**Figure 2:**
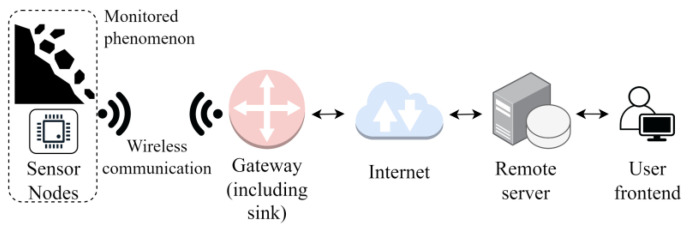
General architecture of an IoT WSN.

The sink unit can be used to monitor the information locally or remotely, if it is connected to another network. In this second case, the internet is the commonly used structure, so to form an Internet of Things [27,28] sensor network. The nodes can be stationary or moving and can be heterogeneous in sensor type and communication technology. Figure 2 shows the general architecture of an IoT-enabled WSN in the example case of rockfall monitoring.

The application fields of WSNs are numerous and, ideally, unlimited. Every application demands specific requirements, and it is the designer who selects the appropriate technological solution for the case. We found several application fields in the literature and industrial fields, such as environmental monitoring [29], structural monitoring [30], and health-related applications [31], including wearable systems [32], early warning tools [33,34], and industrial control [35]. These deployments benefit from WSN applications, especially concerning installation accessibility and wiring constraint reduction [36], along with the possibility of an increase in overall cost effectiveness. New applications are enabled by WSNs, but on the other hand, the design is affected by several constraints that must be taken into consideration [37,38]. Sensing, processing, and communication usually take place using a limited amount of energy. For this reason, the design approach requires particular attention to data processing and communication protocols [39]. Power constraints are crucial for sensor nodes. In some cases, the nodes are deployed in areas difficult to reach, so it is not possible to recharge or replace the battery of the system. In this case, it is necessary to employ techniques to reduce the power consumption of the nodes to improve the lifetime of the WSN [40]. To this extent, energy harvesting techniques can be applied to WSN nodes to obtain recharge mechanisms [41]. Wireless transmission technology is strictly related to the power performances of the nodes. The current trend is to employ low power wide area network (LPWAN) [42,43] solutions which allow, in many cases, good coverage and energy performance to be achieved [44,45]. This aspect is particularly crucial in harsh environments, both in terms of radio propagation and power supply [37]. LPWANs are often used in mixed-type systems, where several different solutions are used in combination [46,47]. Security in a WSN, for topology, is strictly application-dependent, and the sensor nodes should be robust to physical damage and to communication issues such as data leakage and attacks. Detailed aspects of the security of WSNs were thoroughly investigated in [48,49,50]. The fundamental performance limits of WSNs were analyzed in depth in [51], where the authors studied the constraints that affect these structures. 

In Table 1, we find a summary of the pros and cons of the aspects previously discussed about WSNs.

## 3. Rockfall and Landslide Monitoring Based on WSNs

In this section, after a brief overview of other hazard identification and monitoring techniques, the monitoring systems for landslides and rockfalls based on WSNs technology are reviewed, along with some wired implementations of sensor networks dealing with the geohazards in question.

### 3.1. Examples of Non-WSN-Based Monitoring Systems

WSNs are not the only possible method for monitoring rockfalls and landslides. Generally, we can identify two main categories of monitoring methods: remote sensing and ground-based methods. 

Synthetic-aperture radar (SAR) and interferometric synthetic-aperture radar (InSAR) are used to achieve models with accuracy up to a millimetric scale [52]. Infrared thermography (IRT) remote sensing methodology is based on the measurement of the radiant temperature of a surface from a long distance and can provide important parameters such as temperature maps, emissivity, and the humidity of an area. Those are all useful in landslide and rockfall prediction systems [53]. Other physical quantities like temporal displacement, soil moisture content, and type of material may be measured with high resolution using the terrestrial laser scanning (TLS) sensing technique [54,55,56]. The usage of unmanned aerial vehicles (UAVs) has been studied in the literature in many applications. We also found contributions to natural disaster prevention, as the authors of [57] report. These devices can be successfully employed in management strategies for evacuation plans and in risk assessment, but as suggested, UAVs can be used in coverage enhancement for wireless networks. The use of optical very high-resolution (VHR) data in conjunction with analytical methods enables monitoring systems to discriminate between different water contents and vegetation covers [58].

Satellite interferometry [59] and laser scanning are other technologies used for remote land-movement monitoring [60,61]. These solutions offer good performance for movement assessment but, in some cases, are not accessible and have moderate costs. Geographic information system (GIS) data can be used for a preventive risk assessment. In [62], a best-fit method was used to generate the probability maps for landslides by combining different GIS data layers.

Optical fiber (OF) can be used locally for geohazard monitoring in strain and temperature sensing. The possibilities include underground deployment [63] and overground cable installations [64]. The accuracy assessment shows that the models obtained using OF could detect potential rockfalls with an accuracy greater than 90 percent.

**Figure 3:**
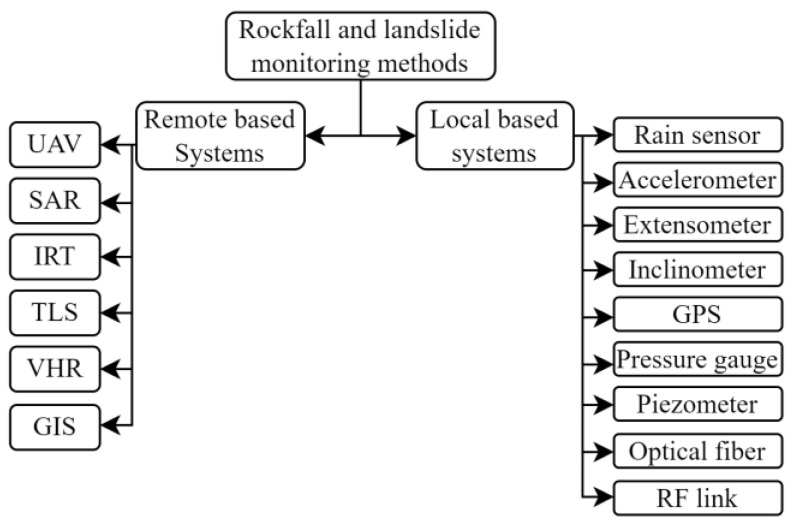
Monitoring mechanisms for rockfall and landslides.

Sensory systems can be developed locally in an area subject to risk using inexpensive instrumentation, also in wired implementation. In [65], a low-cost infrastructure wired-sensor network was presented. The nodes were accelerometer-based and acted as a warning when predetermined acceleration values were exceeded. A similar hybrid wireless–wired system for rock collapse forecasting was presented in [66], where a controlled area network (CAN) bus was used for wired communication with a base station and Wi-Fi [67] for communication between the base station and a control room. Sensor-less networks were also studied to assess if the soil conditions could be analyzed for early warning purposes using the reception parameters of a radio transmission [68], as those vary with the characteristics of the soil in sub-ground installations. The diagram in Figure 3 shows a list of remote and local monitoring systems for landslide and rockfall geohazards.

Structural health monitoring (SHM) is a connected aspect of geohazard events. The authors of [69] reported a review of WSNs applied to SHM. Several works were investigated in detail regarding the hardware architecture, in particular, that of the sensing interfaces and radio communication units. A more recent review was reported in [70], where other insights were studied, such as micro-electro-mechanical systems (MEMS), artificial intelligence (AI) employment, and cloud computing. In [71], the authors showed how the application of SHM basic concepts and scalable wireless networks of sensors could be applied to the monitoring of natural disasters.

### 3.2. WSN-Based Monitoring Systems

In the past, researchers have carried out surveys on warning systems for geohazards [23,72,73], analyzing several works and methodologies available in the state of the art. However, new systems have been developed in recent years.

Early prototype systems were proven to be able to gather useful information using relatively low-cost hardware using microcontrollers, commercial sensors, and low-power wireless transmitters [74]. In [75], the authors use a camera-based system and deep learning to identify the locations of possible landslides in a mountainous region of Thailand. The sensor nodes that were deployed used ZigBee [76] and Long Range (LoRa) [77,78] low-power radio technologies. The authors aimed to investigate techniques that could be used to generate a representative image of daily slope condition from many images captured in one day. To this extent, different data fusion methods were taken into consideration.

MEMS sensors are cheap and can successfully be used to detect movements on rock or soil slopes, as many research studies successfully report. 

A disaster prevention system based on multiple radio technologies was tested in a sloped area around Taiwan in [79]. The equipment was installed on the protection structure, aiming to display the quantitative data of rockfall events. The nodes used accelerometers and wired vibration sensors. The prototypes were built on commercial electronic circuits and encapsulated in plastic enclosures. We could distinguish three types of nodes based on transmission technology. The first was based on LoRa, while the second used narrowband-IoT (NB-IoT) [80] and the third exploited long term evolution (LTE) M1 [81,82]. The LoRa unit communicated data to a Raspberry Pi [83] unit, which transmitted over Wi-Fi to a MySQL [84] server. The barriers can offer differing sensitivities to rockfall with respect to the observation point; therefore, the placement of the sensors is crucial. In the implementation of this system, the authors had to choose appropriate anchorage locations for the nodes on the steel barriers.

The inertial measurement unit (IMU)-based WSNs are used to measure the accelerations and rotations of the installation frame. Based on this, there have been important research efforts made to distinguish the different types of slope movement.

In [85], the authors presented a sensing system to recognize a rock fall event and to monitor the structural parameters of a protection mesh or concrete barrier. The architecture was divided into blocks, organized with a central LoRaWAN [86] gateway unit and sensing nodes disposed on the installation site. The elements gathered information about the vibrations and the relative inclination of the protection barrier using MEMS accelerometers and transmitted them to the sink using the LoRa modulation technique. All the data were collected on a web server used for storage and automatic alerting through messages in the case of critical events. A dashboard showed the summary reports and the collected data. The system is currently active in Pantelleria, Sicily, Italy. The paper describes in detail the hardware and firmware structure of the WSN. The authors faced difficulties in the availability of radio coverage for all the nodes, which, in the case of some elements of the network, gave poor results.

Sensor fusion is the main topic in [87] where the authors presented a WSN for rock mass movement monitoring in Aachen, Germany. Tilt sensor data wads fused with MEMS accelerometer data to detect small rock movements and possible variations in inclination angle. The authors use an ISM band radio communication for the link between nodes and gateway and serial communication for data exchange between the gateway and a control server. The latter was connected to the internet for remote access using GPRS in the outdoor version and using cable in the indoor version. The nodes adopted an adaptive routing pattern to ensure the correct transmission of the packet. If a route is blocked, the nodes will vary the hop sequence for data exchange with the gateway. The MEMS units showed that these sensors have good accuracy and resolution to detect movements like tilting and mass spreading. The sensor fusion allowed the verification of received data, thus, a better interpretation of the dynamics of the affected area. An important point in the SLEWS project was low-cost development with accuracy capabilities. This was accomplished using low-cost sensors with standardized industrial interfaces.

Past works have presented distinct patterns of accelerometric data in the behavior of the structures [88]. Pattern-relative information is fundamental for the understanding of landslide engineering for the implementation of mitigation structures. In [89], the authors presented a system that communicated with an on-site trailer station using the 802.11 g standard [90]. Computers were equipped on the trailer for on-site data analysis and to provide cellular uplink. The latter was used for internet communication to a storage server. The authors carried out experiments using a rock equipped with a sensor device, and the results reported different IMU data for each possible movement pattern of the mass. The authors had to realize a rugged prototype, capable of handling the stress of falling with the rock formation during the tests. Although only small-scale experiments were performed, the results were quite encouraging to justify further studies using this approach in real scenarios.

Received signal strength indicator (RSSI) localization approaches have been deeply studied in the literature [91], and we found several proposals in landslide and rockfall monitoring systems.

In [92], another LoRa-based WSN on an uninhabited hillside was deployed. This area is harsh and affected by to landslides. Therefore, the authors studied the feasibility of a LoRa-based network and focused on the zone coverage of the communication. The evaluation was carried out using RSSI, which was fed to a triangulation algorithm to localize a particular node in the network. If the node had varied its position, then a portion of the landslide had moved. All the elements communicated in a star network to an anchor node where all the data was collected locally. However, the study did not report an in-depth description of the hardware implementation of the sensor nodes and the mounting on the hillside. In order to verify the RSSI model, the authors had to make hundreds of RSSI measurements between one anchor and a sensor for different positions.

The localization of nodes in a landslide using RSSI and the channel state information (CSI) is sensitive to environmental noise. This results in an inaccurately estimated displacement. The authors of [93] applied an information-fusion method based on bounded-error estimation to improve accuracy. The aim of this work was not an actual implementation of a monitoring structure but to provide enhanced localization algorithms for RSSI-based landslide monitoring systems. The experimental results showed that the accuracy was increased by approximately 4.5% compared to conventional methods. 

Another system specifically conceived for harsh environments is described in [94], in which an energy-efficient ad hoc communication protocol was employed to ensure robustness and a long lifetime of the overall system. The network was composed of 15 wireless nodes with sensors of various kinds. One of them was equipped with a GPRS [95] modem to act as a gateway toward the internet. The power supply needs of both the nodes and the gateway were fulfilled with batteries, with the addition of a solar panel in the case of the gateway. This had the disadvantage of requiring periodical human intervention for battery replacement, although the residual battery charge check suggested a worst-case lifetime of more than one year. The results obtained showed quite good power performances with an average power consumption of the node of about 0.192 mW. A custom protocol was developed for this implementation, in order to have reliable communication in a harsh environment. 

In [96], the authors presented a network based on LoRa and intelligent sensing for rainfall and landslide displacement in Shuicheng County, Guizhou Province, China. Four crack nodes were located in cracks to measure the displacement of rock, while one central node acted both as master and gateway. The rainfall was measured at the location of the master node by means of a tipping bucket-switching rain sensor. The hardware and software designs of the system were described comprehensively. The sensor nodes could operate in normal or reduced power mode to obtain a lower current absorption of 1 mA. For this purpose, the authors explained the low-power software design in detail. The sensor nodes operate in slow sampling mode until an over-threshold displacement of 20 mm is sensed. Subsequently, the devices enter a fast sampling mode, collecting data every second until a successful transmission is achieved. The deployed sensor network was able to measure rock movements that exceeded the threshold in one of the test locations, where a sudden displacement was recorded. 

Deep-earth probes are a possible solution for landslide sensing, especially in soils where the presence of rock is reduced. The work presented in [97] dealt with the deployment of one in a region of India. The authors used a single data acquisition board connected to multiple sensors, namely strain gauges, dielectric moisture sensors, piezometers, and a geophone. Some poles were inserted deep into boreholes with a depth of 2 to 20 m. The authors deployed a network containing 49 sensors during this stage of the research. All the sensing elements were read by the node and transmitted to a central gateway using Wi-Fi. The gateway had a satellite link that allowed communication towards a remote server. The power supply to the nodes was achieved by solar harvesting and lead-acid batteries; however, we did not find an analysis of the energy performance. An in-depth description of the sensor placement was reported. The study showed data from 1 month in the period of July–August 2009 that showed the strain gauge deformation and water activity in the bores.

Soil moisture monitoring was also used in [98] where a tensiometer and soil moisture sensors were integrated with an inclinometer. The authors placed three wireless nodes in a landslide-affected area along a hillside in Seoul, Republic of Korea. A comprehensive risk assessment was carried out previous to the installation, in which the authors studied the geomorphological aspects of the area and the vegetation density. The morphology of the environment was also studied for radio propagation purposes because the high density of trees and the height of the test installation were two non-favorable variables for the correct transmission of data. The nodes’ radio layer was based on Wi-Fi standard at 2.4 GHz center band frequency. A sink gateway communicated the data to a remote server using LTE. The system was solar-powered by means of a solar panel and a battery. However, we do not have information about its energy performance. The system allowed the authors to obtain data on the rainfall characteristics at different soil heights for several seasons. The authors compared the results obtained by the real implementation of the WSN with the results that were gathered in laboratory tests. The actual field data showed a lower volumetric content of water due to different soil saturation conditions.

In [99], a LabVIEW program [100] was developed for the purpose of analysis and presentation of the data collected from an energy-efficient WSN that could run unattended for months. The system was built on the IEEE 802.15.4 [101] protocol, and the various monitored parameters were volumetric water content in the soil, seismic vibrations, displacement of the soil layer, soil temperature, and rainfall measurement. The landslide-prone area and hence the nodes used to monitor it were divided into two clusters. A so-called cluster head was designated for each area. The purpose of the cluster head was to aggregate data from all nodes of the cluster area and to transmit data to a gateway node. Finally, through the gateway, collected data were transferred to the data analysis center by using GPRS.

Transport pathways are particularly at risk during a landslide. A WSN for practical use on expressways was developed in [102] in light of the rise in unexpected heavy rainfall events. The system adopted a monitoring station and multiple sensor nodes with several sensing elements. Data measured by the sensors were sent to the monitoring station that aggregated all the arriving data and periodically transferred it to a web server from which they could be downloaded. When different monitoring sites were established, the readings of each were automatically registered on the web server for monitoring the conditions of multiple slopes over the internet. The authors conducted several communication tests to test and improve the robustness of the system. A trial environment was established before the deployment of the full network.

ZigBee WSNs are often used in the field of geohazard monitoring. In [103], the authors presented the hardware design of a system based on ZICM2410 [104] modules. The nodes were built to be mounted on a slope and were equipped with a tilt sensor, temperature and moisture sensors, an extensometer rod for displacement, and a capacitive rainfall sensor. A remote management unit was connected using GSM to a base station that gathered the nodes’ data. A low power schedule of the working cycles was used to have better management of the battery power: the nodes sleep for several hours and have an active period of about 1 min where the power is increased. We do not have information of whether the system was deployed in a real scenario; however, the authors focused on the description of this low-cost solution for the sensor nodes.

Similar hardware as the aforementioned system is found in [105], where the authors focused on the study of the hardware performances of a monitoring system that was installed in Guizhou, China. The system has been proven able to work consistently for a long period of time in a harsh environment.

In [106], the authors presented a smart landslide monitoring device called “SMARTMODE”, which could be inserted into a WSN scenario. The hardware consists of a microcontroller, a communication module, general-purpose input–output (GPIO) channels, and power circuitry. In particular, the node can be used in a ZigBee-based WSN, and is supplied using rechargeable batteries. The sensor components on the device were connected through plug-and-play connectors to allow the faulty parts to be replaced. SMARTMODE monitored the slope displacement using a MEMS-based three-axis in-place inclinometer (IPI). The slope-related parameters, which are soil moisture and soil pore-pressure (PP) were also monitored using capacitive sensors and pressure sensors. The software was responsible for data aggregation, context-adaptive operations, and energy optimization of the SMARTMODE. One of the software features was the filtering of the sensor data from noise and outliers to prevent false warnings. The authors faced one common difficulty that comes with soil monitoring, as was observed in previously mentioned research. Soil moisture sensors are sensitive to changes in soil properties; therefore, it can become difficult to decide whether a small change in the read values is due to rain or environmental fluctuations. This can happen until a significant change in sensing values has been observed.

It is known in research that clustering helps to increase a network’s lifetime and its routing performance. To this end, in [107], an autonomous structural monitoring system for dynamic events was presented. The system was composed of nodes capable of point-to-multi-point transmission to form a redundant routing pattern. This was needed due to harsh environmental conditions because the network was deployed on rockfall barriers. The nodes were equipped with an accelerometer for monitoring dynamic activity and variations in the inclination of the mounting barrier, and all the units communicated using a 433 MHz ISM radio module. The elements were battery-powered, and solar harvesting was used to achieve energetic sufficiency. A unit in the network could be configured as a bridge or repeater while retaining the sensing capabilities. A gateway towards the Internet was always listening for transmissions of accelerometric data and forwarded the received information using GSM. All the systems were described at the hardware and functionality level, and real scenario test results were reported. One of the main difficulties that the authors faced was the poor radio coverage of the zone where the system was developed. To this extent, a mixed mesh was used as network topology.

A first approach to radio frequency identification (RFID)-based land movement monitoring was proposed in [108]. The authors applied the principle of phase unwrapping to observe the variation in the phase of a response signal to the interrogation of the tags. A base station was responsible for the interrogation, which produced an RFID response used to process the radial distance between the system origin and the single tag location. A dense network of tags, together with multiple frequency scanning were the key factors to enhance the accuracy of phase unwrapping methods [109]. The aforementioned study included the RFID data from four landslides that share the same measurement scheme. Several RFID tags were read by an acquisition system consisting of an interrogator and at least two reader antennas. The measurement rate depended on the available power and on the site. The autonomous stations relied on renewable energy and used a lower acquisition frequency with respect to power-grid supplied stations. The data reported a minimum of 100 phase readings per tag every day. Both the phase of arrival (PoA) and the received signal strength (RSSI) were measured. The scenario of a difficult tag reading due strong multipath interference or poor signal strength was one of the main difficulties of this work. To overcome this problem, the authors applied a data fusion approach, obtaining a data availability increase.

Multi-technological monitoring approaches can be beneficial for risk assessment. In [110], the authors presented a mixed system located in the Montserrat Massif, Catalonia, Spain. A large rocky wall is located above an inhabited area, posing a threat to the population. Therefore, the researchers installed a WSN based on extensometers to monitor the movements of the rock masses. These devices enabled precision tracking, while larger area monitoring was carried out using radar scanners. This second method was employed both in the aforementioned location and in a second unpopulated section of the mountain chain. The information obtained with the local and radar sensing was integrated with temperature measurements to obtain the relationship between the displacement events and the environmental causes. One of the main difficulties that the authors encountered in the mentioned work was related to the atmospheric changes that resulted in a distortion of the interferometric phase measured using the SAR system. These effects are particularly strong in mountainous areas due to the rapid variability of weather conditions.

In [111], the development of slope and river monitoring systems employing three types of LPWANs was reported. In particular, the installation included a total of 18 tilt sensors, 6 extensometers, an ultrasonic river water depth sensor, and a temperature and humidity sensor to carry out monitoring at selected observation sites in Japan (Nakayama, Aruse, Namikata, and Matsuyama). The network made use of combined technologies because two units used Sigfox, and LTE-M, while the rest communicated over LoRa technology. Batteries were used to power all the nodes, in conjunction with solar panels for the ultrasonic one to recharge the battery. This means that periodic batteries replacement is required. Emphasis was placed mainly on three aspects of the WSN. First, the determination of the proper locations for the gateway and the sensors, based on experimental RSSI, SNR, and PRR (packet reception ration) values. Second, the selection of the phenomena to be monitored and of the type of sensor for each quantity to be measured. Third, the role of temperature on the measured inclination. During the experimental stage, the authors reported the occurrence of errors from the sensors for several reasons such as electrical problems, reduced battery lifespan and temperature and humidity effects. These problems created difficulties in the study, which led to the replacement of a part of the devices in one site.

Table 2 reports a summary of the presented research works. The stage represents the status of the considered WSN, as in some cases, the works present only prototypal results or deal with fixed installations that are located in a geologic area for a longer time. The monitored element reports the particular type of structure and event that is monitored by each system. Table 3 provides geographical and geological information concerning the location of the research study, and witnessed events.

Finally, Table 4 reports some details about the considered monitoring systems. All the fields are relative to the sensor nodes of the WSN and not to elements that come after the gateways. The low power performances are relative to the lowest current absorption that a sensing node draws from the battery. This is achieved, in most of the works, when the nodes enter a standby mode between each data gathering and transmission cycle. Unfortunately, not all the research works provide all the information regarding hardware description and power performances.

## 4. Considerations on the State of the Art and Future Perspectives

The listed and commented research works highlight several key factors. As other studies previously reported, WSNs for geohazard monitoring follow the current technological trend of LPWAN implementation [116]. The optimal energy performance of these network solutions certainly plays a key role in their adoption.

The reviewed works suggest that MEMS sensors result in being a widely adopted choice, as these are usually inexpensive and come in small packages, thus favoring small node dimensions. Moreover, these circuits usually have low power characteristics, as reported in many of the cited works. The accelerometric and tilt sensors are more widely used in landslide monitoring, as there is a need to observe any variation in the spatial position of the nodes. In rock mass monitoring, extensometers are a choice that is often adopted, as in some cases, the falling patterns of rocks are predictable. Using extension rods, the researchers can easily execute a quantitative risk assessment by evaluating the rock displacement. In muddy environments with low-speed debris flow, water content is the central monitored element. Solar harvesting has resulted as the solution for energetic management that is more often used in geohazard monitoring, being a cheap solution both in terms of cost and space requirements. The energetic requirements for the sensor nodes in the analyzed research works are various. From the available information, we observed that the power dissipation in all the configurations of the sensing devices ranges from the micro Watt during the idle state to the Watt during transmission intervals. In standby mode, most of the systems reported a power dissipation in the range of milliwatts. Two types of battery were the most chosen for the nodes: lithium and lead-acid types, possibly due to the low cost.

**Figure 4:**
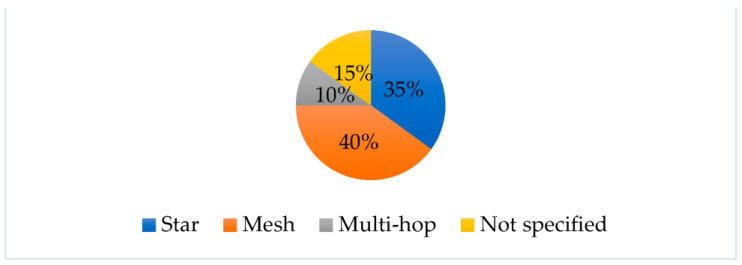
Network topologies pie chart for the considered works.

The network topologies for the considered works reported a major use of mesh and star structures, as we can observe in the pie chart of Figure 4.

The mesh topology seems to offer robust communication performance and reliability in terms of packet delivery. The star topology was adopted in many works as the nodes do not have particular wireless connectivity issues. Multi-hop routing was shown to be useful in cases where network coverage was particularly difficult to achieve.

**Figure 5:**
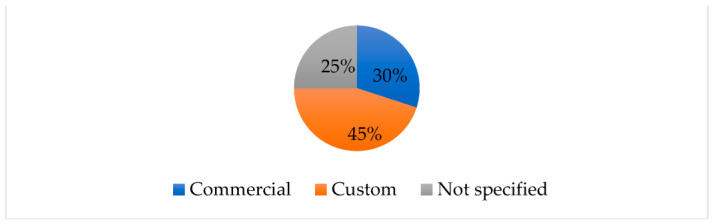
Hardware platform pie chart for the considered works.

Most of the reviewed works made use of custom hardware for node implementation, as reported in Figure 5. These elements are usually composed of a radio unit and sensor interfaces, all managed by a central microcontroller unit. The commercial hardware platforms were also often used, in particular for laboratory prototypes. Some of the considered works did not report particular information about the employed hardware, as, in some cases, it was not the focus of the work.

**Figure 6:**
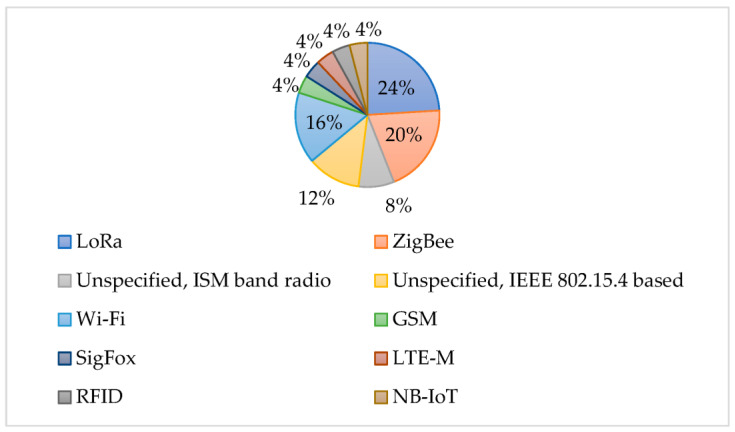
Communication technologies pie chart for the considered works.

Figure 6 shows a pie chart reporting the communication technologies adopted in the considered works. LoRa emerged as a widely adopted technology among all the reviewed works. This was due to its good power performance [117], and the possibility of a free media access control layer (MAC) [118]. ZigBee was used almost equally, possibly for its good power performance and network structure flexibility.

Several challenges can be identified in the reported works as common difficulties in the development of the systems. The energetic sufficiency of the nodes that compose the networks is a fundamental aspect. As we observed, many of the implementations used energy harvesting techniques. Finding an optimal network topology is a widely studied task in WSN structures, as previous studies report [119,120]. The reviewed research suggested that for geotechnical-monitoring WSNs, the most commonly used topology is the star.

The miniaturization of wireless nodes represents a future perspective for sensor networks [121]. Currently, most of the networks are built using commercial standalone components that incur low overall cost. The current miniaturized solutions for sensor networks require ad hoc solutions that are, in most cases, not yet industrialized [122,123]. The harvesting technique employed in geohazard monitoring can surely benefit from miniaturized solar cells [124] to reduce the nodes’ dimensions. The employment of machine learning and artificial intelligence has various application possibilities in geohazard monitoring and is currently a very studied field [125]. The predictive algorithms help to enhance susceptibility models [126] and are useful for the differentiation of warning events based on the movement patterns recorded by the nodes. More capillary integration of AI edge computing in networks, like those that have been reported, would be an effective tool for risk assessment and manutention operations.

## 5. Conclusions

Climate change and urbanization increase the risk of geohazards in the near future. For this reason, it is necessary to develop monitoring systems for hazard prevention and management. Historically, techniques of remote sensing have been used to observe deformation in geologic formations. However, these systems can be expensive. The development of wireless sensor network solutions opens up new possibilities for local monitoring at low cost.

This study reviewed the monitoring systems for landslides and rockfall events based on WSN. An analysis of the different monitoring systems was carried out, reporting implementation materials and methods, along with the functionality. Geographical and geotechnical details of the implementations were also reported. Some of the main characteristics of the reviewed research works were summarized.

We can observe that the Internet of Things approach is the preferred choice for the more recent systems, as it allows flexibility. An analysis of the electronic components showed the different types of sensors used in different types of land movements, reporting a wide spectrum of sensor types for different applications. The LPWAN technologies were defined as the most frequent choice for radio communication, as they allow low-power performance by the sensor nodes. The majority of the systems present energy management techniques based on standby modes to achieve reduced current absorption from the battery.

The current trends, achievements, and problems of the topic under investigation were discussed, along with future possibilities.

## Figures and Tables

**Table 1:** Pros and cons of WSNs.

Pros	Cons
Energy efficiency	Limited range (relative to the application)
Flexibility and scalability	Some implementations can be subject to interference
Installation accessibility	Security
Wiring constraint reduction	Power constraints
Cost effectiveness	Possible data amount and speed limitations

**Table 2:** Year, system stage, monitored hazards, and study duration.

Ref.	Year	Stage	Monitored Elements	Study Duration
[74]	2012	Laboratory prototype	Soil condition	Not specified
[75]	2021	Fixed installation	Land displacement	Not specified
[79]	2022	Fixed installation	Rockfall mesh barriers	1 day
[85]	2022	Fixed installation	Rockfall mesh Concrete barriers Environmental parameters	July 2022—to date
[87]	2009	Fixed installation	Rock mass tilting	Not specified
[89]	2019	Portable test nodes	Rockfall pattern	Not specified
[92]	2017	Not specified	Hillside landslide movement	Not specified
[94]	2016	Fixed installation	Land displacement Environmental parameters	9 months
[96]	2022	Fixed installation	Crack displacement Rainfall	9 months
[97]	2011	Fixed installation	Deep earth movements Hydric characteristics	Not specified
[98]	2020	Fixed installation	Soil moisture status	Not specified
[99]	2016	Fixed installation	Seismic movements Soil moisture	Not specified
[102]	2018	Fixed installation	Rain-induced slope failure Sediment disasters near expressways	1 month
[103]	2011	Laboratory prototype	Soil displacement	Not specified
[105]	2017	Fixed installation	Large crack displacement Rainfall	Not specified
[106]	2023	Fixed installation	Land movements Hydric characteristics	Not specified
[107]	2018	Fixed installation	Rockfall barrier movements	10 months
[108]	2023	Fixed installation	Land displacement	2 years
[110]	2016	Fixed installation sensor nodes, fixed and portable radar scanners	Rock mass movements	3136 days
[111]	2018	Fixed installation	Slope and river monitoring	3 years

**Table 3:** Region of study, country, type of monitored geohazard.

Ref.	Region	Country	Witnessed Events
[74]	Not applicable	Not applicable (laboratory prototype)	Not applicable
[75]	Northern Thailand	Thailand	Not specified
[79]	Nantou	Taiwan	Rockfalls disasters
[85]	Pantelleria	Italy	No significant rockfall events in the reported period
[87]	Not specified	Not specified	A test was performed showing tilt monitoring capabilities
[89]	Not applicable	Not applicable	Not applicable
[92]	Not specified	Not specified	Not specified
[94]	Torgiovannetto	Italy	No events in the reported period
[96]	Jianshanying disaster area, Shuicheng County, Guizhou Province	China	Landslide rainfall, landslide deformation and cracks
[97]	Anthoniar Colony hill, Munnar town, Idduki District, Kerala State	India	Not specified
[98]	Seoul	South Korea	Landslides in urban areas, increased heavy rainfall
[99]	Malin village, Pune Maharashtra town	India	Landslide occurred in July 2014
[102]	Not specified	Japan	Slope failures
[103]	Not applicable	Not applicable	Not applicable
[105]	Hezhang County, Guizhou Province	China	Not specified
[106]	Netala landslide location, Garhwal Himalayan Uttarakhand region, Uttarkashi district	India	Landslide occurred during the monitoring period
[107]	Two unspecified sites in south and central Italy.	Italy	No significant rockfall events in the reported period
[108]	Four different locations: - Bidart landslide location on the south-east coast of France, - Harmalière landslide location (Sinard, France) near Grenoble in the western Pre-Alps, - Pont-Bourquin landslide location the western Pre-Alps near Lausanne in Switzerland, - Valloire landslide location in a steep valley (Beaujournal) above the city of Valloire (France)	France and Switzerland	Landslides occurred in each selected site
[110]	Montserrat Mountain, near Barcelona, Catalonia, northeast of Spain	Spain	Rockfalls detached between 2001 and 2008
[111]	Nakayama, Aruse, Namikata and Matsuyama sites	Japan	Increased heavy rainfall events reported

**Table 4:** Technical parameters of the reviewed WSNs.

Ref.	System Monitoring Type	Sensor Types	Node Communication Technology	Node Power Supply	Low Power Performances	Network Topology	Hardware Platform
[74]	Local	Soil moisture sensor, pressure sensor, flexible band	IEEE 802.15.4 based unspecified protocol	AA batteries	Not specified	Not specified	Crossbow MicaZ [112]
[75]	Remote	Cameras	ZigBee, LoRa	Not specified	Not specified	Star	Not specified
[79]	Remote	Accelerometer, vibration gauge, temperature, soil resistance	LoRa, NB-IoT, Wi-Fi	Not specified	Not specified	Not specified	Arduino e Mega 2560 [113] and Pro Mini [114] boards with commercial modules
[85]	Remote	Accelerometer, temperature, humidity, altitude	LoRa	Solar harvesting, 3.7 V lithium battery	16 µA sleep mode current	Star	Custom
[87]	Remote	Accelerometer, tilt sensor	Unspecified 868 MHz ISM radio	Solar harvesting, unspecified battery	Not specified	Multi-hop	Custom
[89]	Remote	IMU	Wi-Fi	Solar harvesting, 3.7 V lithium battery	50 mA in passive mode	Mesh	Raspberry Pi- based [83] node
[92]	Local	IMU, altitude	LoRa	Not specified	Not specified	Mesh	Custom
[94]	Remote	Extensometers, hygrometers, clinometers, thermometer, rain and wind gauges	IEEE 802.15.4 based unspecified protocol	Solar harvesting, 6 V lead-acid battery	30 µA sleep mode current	Multi-hop	WinetTX board [115]
[96]	Remote	Tipping bucket, extensometer, tilt sensor	LoRa	Solar harvesting, 12 V lithium battery	1 mA sleep mode current	Star	Custom
[97]	Remote	Geophone, moisture sensors, strain gauges, piezometers, rainfall sensor	Wi-Fi	Solar harvesting, lead-acid battery	Not specified	Not specified	Custom
[98]	Remote	Soil moisture sensors, tensiometer, inclinometer	Wi-Fi	Solar harvesting, unspecified battery	Not specified	Mesh	Not specified
[99]	Remote	Dielectric moisture sensors, accelerometers, tilt sensors, temperature sensors, rain gauge sensor	IEEE 802.15.4 based unspecified protocol	AA batteries	Not specified	Mesh	Not specified
[102]	Remote	Tilt sensors, extensometers, displacement meter, soil moisture sensor, water gauge, rain gauge, temperature sensor	ZigBee	Solar harvesting, 3.6 V rechargeable batteries	<1 uA sleep mode current	Mesh	Custom
[103]	Remote	Extensometer, tilt sensor, capacitive rainfall sensor, temperature, and moisture sensor	ZigBee	Unspecified lithium battery	1 mA sleep mode current	Mesh	ZICM2410 ZigBee Module with commercial devices
[105]	Remote	Rainfall sensor, tilt sensor, displacement sensor	GSM	Solar harvesting, unspecified battery	Not specified	Star	Custom
[106]	Remote	IMU, moisture sensor, pressure sensor	ZigBee	Solar harvesting, 3.7 V lithium battery	34 mA in sleep mode	Star	Commercial devices on a connection board
[107]	Remote	Accelerometer	Unspecified 433 MHz ISM radio	Solar harvesting, 3.7 V lithium battery	3 mA sleep mode current	Partial mesh	Custom
[108]	Not specified	RFID tags	RFID	Power grid, Solar harvesting and wind harvesting for the interrogators	Not specified	Star	Not specified
[110]	Remote sensor nodes, ground-based SARs	Extensometers, temperature sensors, radars	ZigBee	Solar harvesting, rechargeable AA batteries	Not specified	Mesh	Custom SAR equipment
[111]	Remote	tilt sensors, extensometers, ultrasonic sensor, temperature and humidity	LoRa, Sigfox, LTE-M	Unspecified alkaline batteries	Not specified	Star	Not specified

## Data Availability

No new data were created or analyzed in this study. Data sharing is not applicable to this article.
